# Lecture on Displacements of the Uterus

**Published:** 1839-06-01

**Authors:** W. Harris

**Affiliations:** Philadelphia Medical Institute


### CLINICAL LECTURE.

### LECTURE ON DISPLACEMENTS OF THE UTERUS, *delivered by* W. Harris, M. D., *at the Philadelphia Medical Institute.*

#### RETROVERSION OF THE UTERUS.

Retroversion of the uterus, is a displacement of rare occurrence. The young physician should, nevertheless, be familiar with its character and treatment.

The fundus of the uterus, in retroversion, falls backwards into the hollow of the sacrum, making pressure against the rectum, while the os tincæ and cervix rise up above the symphysis pubes. The inclination backwards may take place in different degrees; but in every case of complete retroversion, the fundus will be found under the sacro-vertebral angle, in the concavity of the sacrum.

This displacement may take place either in the unimpregnated uterus, or during the gestative process of that organ.

#### CAUSES OF RETROVERSION.

The causes which produce retroversion, are a preternaturally large pelvis, an over-distended bladder, violent vomiting, great physical exertion, blows, falls, &c.

The overturn of the fundus may take place either suddenly, or very slowly, according to the greater or less violence of the impulse that produces it. And the symptoms, as to their intensity, will be influenced by the condition of the uterus at the time it takes place. If the womb be impregnated, and the displacement occurs suddenly, unless promptly relieved, the symptoms will become violent and alarming. The flow of urine will be obstructed, and the fœcal passage interrupted. Periodical pains of a bearing down character may come on, threatening abortion. Faintness, and sometimes vomiting, accompany this mal-position. In the retroversion of the unimpregnated uterus, the symptoms are similar to those of the impregnated, but less intense, and the evils arising from it less urgent. And, in this case, if the uterus be allowed to remain in its mal-position, the contiguous parts will accommodate themselves, in time, to the displaced organ, but will never become entirely reconciled to the obtruder.

When the physician finds that the symptoms above enumerated are present, he should proceed, without delay, to a per vaginam examination, when he will find, if a retroversion has taken place, a round tumour in the posterior part of the lower pelvis, pressing against the concavity of the sacrum. This tumour will be small if the womb is of its natural size; but, if impregnated, its dimensions will be greater or less, according to the age of the fœtus. The operator should, at the same time, with a view to a more perfect diagnosis, pass his finger up behind the symphysis pubis, where he will be able to feel the os tincæ, unless the neck has mounted up so high as to place the mouth beyond his reach.

The diagnosis between retroverted and prolapsed uterus is not difficult; as in the former the os tincæ and cervix are out of reach, while in the latter they are both readily felt. Besides, in the former, the organ is firmly and obstinately fixed, while in the latter it is light and moveable.

The diagnosis, too, between the retroverted uterus and an enlarged ovary, or an extra-uterine pregnancy, when either may chance to occupy the posterior part of the pelvis, is readily made, by the operator being able, in the latter cases, to feel the os tincæ, while in the former it is above the symphysis pubis. The same rule will prevent retroversion being confounded with a serous or hydated cyst, which is sometimes developed between the vagina and rectum.

The diagnosis between retroversion and some of the diseases just enumerated, especially extra-uterine pregnancy, enlarged ovary, and hydatids, is sometimes exceedingly difficult,—so much so, indeed, that some of the most distinguished accoucheurs and surgeons of Europe have not been able to distinguish the one from the other.

#### THE TREATMMENT OF RETROVERSION.

If the displacement has existed for years, and its character been misunderstood, the reduction may be very difficult, and even impossible, owing to adhesions having taken place. Happily, this case of retroversion occasions but little inconvenience. The only thing necessary to be done, is to take care that neither the urinary nor fœcal passage becomes obstructed.

But, if the uterus be impregnated, and retroversion take place suddenly, the first thing that requires the physician's care, is the suppression of the urine. Fearful consequences have resulted, even the bursting of the bladder, from this circumstance having been too long neglected.

The next object requiring attention, is the state of the rectum, which is, in many cases, blocked up with fœces. To remove which, castor oil may be administered by the mouth, and its operation assisted by an enema. But if this fail, owing to the fœces being impacted, as is sometimes the case, the bullet scoop, or the index finger, must be used to rake out the rectum. Having evacuated the bladder and rectum, if there be no inflammation in the parts, the operator should proceed at once to the restoration of the displaced organ.

To accomplish this, the patient should be placed upon her back, with her breech projecting over the edge of the bed, her thighs and legs bent, and her feet resting upon two chairs. The operator's right hand, having been previously anointed with lard, should be introduced in a state of supination, into the vagina, the back part of the fingers towards the sacrum, by which the base of the tumour should be pushed up along its curve until it rises above the promontory. The pressure must sometimes be severe and long continued, before the restoration is accomplished. If this manœuvre fail, the operator should next introduce his index finger into the rectum, as, by making pressure through that channel upon the *fundus uteri*, he may eventually succeed. It is proposed by Madam Boivin to divide the sphincter ani, or to dilate it by the extract of belladonna, that the operator may be enabled to introduce the hand into the rectum; but this, it appears, is unnecessary, as Dr. Parent succeeded, without either of the above proposed means, in introducing his whole hand into the rectum,—and Dr. Dusaussois had previously accomplished the same feat, and this, too, as they both represent, without causing any considerable pain. Failing, however, the patient should be put in a warm bath, and afterwards bled freely from the arm, when the restoration should again be attempted. Dr. Dewees, in such cases, sometimes bled *ad deliquium animi*. He says the greatest difficulty he had to contend with in the restoration, in ordinary cases, was the involuntary bearing down efforts of the woman, which were provoked by the presence of the hand in the vagina. To overcome this, his practice was to bleed the patient in a standing position *ad deliquium*, and, while in a state of syncope, to place her suddenly upon her back in bed, and proceed, with the least possible delay, to restore the displaced organ. Failing to succeed with the patient placed upon her back, she should get upon her knees and elbows, when the effort may succeed better. The prone position is often the more convenient for the replacement of the fundus uteri, especially as its own weight, in this attitude, favours its restoration. Besides, the operator can now more easily introduce his fingers into the rectum, to push back the base of the tumour. It may be necessary, at the same time, to introduce one or two fingers into the vagina, behind the pubes, or a strong sound into the bladder, for the purpose of drawing down the os uteri, whilst the fundus is pushed up. This twofold motion has succeeded, after the elevation, singly, has been unsuccessful.

The restoration being accomplished, if the uterus is unimpregnated, a pessary, of appropriate dimensions, should be introduced, to prevent a redevelopment, when the patient may return to her ordinary avocations.

As the pessaries, hitherto contrived, have failed to fill all the indications, and the displacement occasionally to return, Dr. Moreau declares in his "Traité Pratique des Accouchemens" that, by a pessary of his own invention, which is made exactly to fit the concavity of the sacrum, and extend from the cul de sac upon the posterior face of the cervix uteri to the perineum, he has treated a number of cases with complete success. That the precise manner in which it is constructed may be understood, I quote his own words:—"We then took a piece of cork about five inches long, and a square inch thick; we so shaped it that it presented a convexity behind, and a slight concavity in front; its angles were rounded off, its lower extremity cut obliquely from before backwards, its upper in the same direction, but so as to take off the anterior two-thirds of its thickness to the extent of more than an inch, in order not to interfere with the neck of the uterus, or press upon it in this direction, and so as to leave on the posterior plane a tongue four or five lines thick, and eight or ten long. We perforated it in its whole length with a red-hot iron, so as to allow the escape of the secretions of the uterus. This shapeless pessary was covered with white wax, and placed in the vagina."

I should be gratified if one of the gentlemen of the class would make, from this description, a pessary, after Professor Moreau's model, and present it to one of the instrument makers of this city as a pattern, for the benefit of the profession.

The restoration of the displaced uterus, if pregnant, should be accomplished, if possible, before the end of the fourth month of gestation, as, soon after that period, the fœtus would be too large to be pushed up through the superior strait. If, however, the case shall have been too long neglected to admit of restoration, what is to be done? Labatier recommends, if the urinary passage be obstructed, to puncture the bladder; but this is unnecessary, as Burns observes—"I cannot conceive of any case where a gum catheter could not be introduced; I certainly have never met under any circumstances with such a difficulty." In this condition, our only hope of a restoration is from bringing on abortion. To accomplish this, the membranes should be ruptured through the os tincæ; but, if this cannot be accomplished, the uterus and membranes, as Hunter advises, should be pierced with a trocar, either through the vagina, or rectum, and after the waters are evacuated, the bulk may be so diminished as to admit of restoration; but, if not, abortion may in this way be brought on, and the fœtus be expelled through the os uteri. This failing, however, the operation of hysterotomy must be performed through the vagina, and the fœtus brought away through that channel.

Purcell and Gardien recommend, for this purpose, a section of the symphysis pubis. But, besides the danger and evil consequences of such an operation, it cannot meet the difficulty; as the principal obstacles to reduction are in the concavity of the sacrum, and at the sacro-vertebral projection.

"Might not the abdomen be opened," says Madam Boivin, "in desperate cases, by an incision in the hypogastrium, and the hand be introduced into the pelvis, in order to raise the uterus?" This could not always succeed, as, in the case examined by Hunter after death, the uterus could not be made to pass through the superior strait, until the bones of the pelvis had been widely separated by means of a saw.

